# The first exome wide association study in Tunisia: identification of candidate loci and pathways with biological relevance for type 2 diabetes

**DOI:** 10.3389/fendo.2023.1293124

**Published:** 2023-12-19

**Authors:** Hamza Dallali, Wided Boukhalfa, Nadia Kheriji, Meriem Fassatoui, Haifa Jmel, Meriem Hechmi, Ismail Gouiza, Mariem Gharbi, Wafa Kammoun, Mehdi Mrad, Marouen Taoueb, Asma Krir, Hajer Trabelsi, Afef Bahlous, Henda Jamoussi, Olfa Messaoud, Abdelmajid Abid, Rym Kefi

**Affiliations:** ^1^ Genetic typing service, Institut Pasteur of Tunis, Tunis, Tunisia; ^2^ Laboratory of Biomedical Genomics and Oncogenetics, Institut Pasteur of Tunis, Tunis, Tunisia; ^3^ University of Tunis El Manar, Tunis, Tunisia; ^4^ Faculty of Medicine of Tunis, University of Tunis El Manar, Tunis, Tunisia; ^5^ MitoLab Team, Unité MitoVasc, UMR CNRS 6015, INSERM U1083, SFR ICAT, University of Angers, Angers, France; ^6^ Laboratory of Clinical Biochemistry and Hormonology, Institut Pasteur of Tunis, Tunis, Tunisia; ^7^ Research Unit on Obesity, Faculty of Medicine of Tunis, Tunis, Tunisia

**Keywords:** type 2 diabetes, exome wide association study, Tunisian population, gene-based analysis, pathway-based analysis

## Abstract

**Introduction:**

Type 2 diabetes (T2D) is a multifactorial disease involving genetic and environmental components. Several genome-wide association studies (GWAS) have been conducted to decipher potential genetic aberrations promoting the onset of this metabolic disorder. These GWAS have identified over 400 associated variants, mostly in the intronic or intergenic regions. Recently, a growing number of exome genotyping or exome sequencing experiments have identified coding variants associated with T2D. Such studies were mainly conducted in European populations, and the few candidate-gene replication studies in North African populations revealed inconsistent results. In the present study, we aimed to discover the coding genetic etiology of T2D in the Tunisian population.

**Methods:**

We carried out a pilot Exome Wide Association Study (EWAS) on 50 Tunisian individuals. Single variant analysis was performed as implemented in PLINK on potentially deleterious coding variants. Subsequently, we applied gene-based and gene-set analyses using MAGMA software to identify genes and pathways associated with T2D. Potential signals were further replicated in an existing large in-silico dataset, involving up to 177116 European individuals.

**Results:**

Our analysis revealed, for the first time, promising associations between T2D and variations in *MYORG* gene, implicated in the skeletal muscle fiber development. Gene-set analysis identified two candidate pathways having nominal associations with T2D in our study samples, namely the positive regulation of neuron apoptotic process and the regulation of mucus secretion. These two pathways are implicated in the neurogenerative alterations and in the inflammatory mechanisms of metabolic diseases. In addition, replication analysis revealed nominal associations of the regulation of beta-cell development and the regulation of peptidase activity pathways with T2D, both in the Tunisian subjects and in the European in-silico dataset.

**Conclusions:**

The present study is the first EWAS to investigate the impact of single genetic variants and their aggregate effects on T2D risk in Africa. The promising disease markers, revealed by our pilot EWAS, will promote the understanding of the T2D pathophysiology in North Africa as well as the discovery of potential treatments.

## 1 Introduction

Type 2 diabetes (T2D) is a multifactorial disease involving genetic and environmental components (1). A large number of Genome-Wide Association Studies (GWAS) have been carried out to investigate its genetic basis. These studies, performed mostly in Europeans, identified more than 240 susceptibility loci comprising over 400 association signals with T2D (2–4). Despite these findings, the understanding of the biological mechanisms of this disease is still limited. In fact, GWAS-derived SNPs always have small effects on complex traits as they can be in high linkage disequilibrium (LD) with the real causal variants. In addition, most of them are located within intronic and intergenic regions, which makes the interpretation of their roles very difficult (5).

In order to overcome these problems, advances in genome sequencing have enabled the investigation of coding variants, providing further functional insights of disease mechanisms. In this context, recent exome genotyping chips and whole exome sequencing (WES) based studies have identified coding variants associated with T2D (6–13).

Many studies have reported differences in LD structure, minor allele frequencies (MAF), as well as effect sizes of several T2D susceptibility variants between different ethnicities (14). Therefore, conducting association studies in underrepresented populations is crucial to uncover novel insights about genetic diseases (15). For instance, Barbitoff et al. identified five susceptibility loci for T2D in the Russian population upon analysis of exome sequencing data of 89 individuals (49 patients with T2D and 40 controls) (6).

T2D is a public health problem in Tunisia, with a prevalence estimation of 23% according to the last national epidemiological study (16). This prevalence is predicted to reach 26.6% by 2027 (17). Hence, urgent measures are needed to better understand population specific risk factors of T2D and to alleviate its burden.

Here, we conducted the first pilot Exome Wide Association study (EWAS) on T2D in the Tunisian population. Our aims were: 1) to identify novel genetic loci associated to T2D in Tunisian subjects; 2) to study the aggregate effects of variants and genes on the predisposition to T2D; 3) to replicate these analyses in a large European in-silico dataset.

## 2 Materials and methods

### 2.1 Study population

This study was carried out in the frame of the ACIP 2017_45 project. The Institutional Review Board at Institut Pasteur of Tunis approved the study (reference number: 2018/08/I/LR11IPT05/V2). It was conducted in accordance with the declaration of Helsinki.

A total of 50 subjects (33 cases with T2D and 17 controls), aged between 35 and 65 years old, were enrolled in the study. T2D was diagnosed based on the American Diabetes Association criteria, including fasting plasma glucose (FPG) ≥ 1,26 g/l and/or glycated hemoglobin A1C (HBA1c) ≥ 6.5% (18). After obtaining written informed consents, all patients were interviewed to obtain their family history of diseases, as well as anthropometric (weight, height, body mass index (BMI=weight (Kg)/height^2^(m^2^))) and clinical data. Subsequently, blood samples were collected for biochemical and molecular analyses.

### 2.2 Whole exome sequencing

DNA samples of all participants were extracted using the Flexigen DNA kit (QIAGEN). The quantity and the quality of the extracted DNA were measured using the DENOVIX DS-11 nanodrop spectrophotometer. Then, WES was performed at Macrogen (South Korea). Briefly, exome capture was carried out using Agilent SureSelect V6, and massive parallel sequencing was then performed on an Illumina Novaseq 6000 system to generate 151-bp paired-end reads.

### 2.3 Bioinformatic analysis

Quality control of the raw sequencing data was performed using the FastQC program (http://www.bioinformatics.babraham.ac.uk/projects/fastqc) with default settings. Then, paired-end reads were mapped to the hg38 human reference genome, and the resulting alignments were prepared for variant calling according to the GATK best practices. Subsequently, genetic variants were called using GATK in a single merged VCF file, followed by an application of variant score quality recalibration using the GATK VQSR algorithm (19). Finally, we used Annovar, implemented in Varaft tool, to annotate the variants for all the samples in the merged VCF file (20, 21). The variants were assigned to genes and functionally annotated with allele frequencies in 1000 Genomes project (https://www.internationalgenome.org/) and GnomAD (https://gnomad.broadinstitute.org/) (22, 23). In addition, we assessed the effects of non-synonymous variants by 12 pathogenicity prediction algorithms: SIFT (24), Polyphen HDIV (25), MutationTaster (26), Mutation Assessor (27), PROVEAN (28), MetaSVM, MetaLR (29), LRT (30), FATHMM (31), DANN (32), CADD (33) and VEST3 (34).

### 2.4 Variant filtration and quality control

In the present study, we focused on autosomal coding variants, including non-synonymous, stop-gain, stop-loss variants and frameshift indels. The impact of the non-synonymous genetic variants was assessed by an approach similar to that developed by Pezzilli et al. (35). It comprises 12 pathogenicity-prediction software packages, that were chosen because of their performance and high classification records (36, 37). The scores generated by these packages were binarized to 1 when the following conditions were met, and to 0 otherwise: SIFT score < 0.05, PolyPhen2 HDIV score > 0.453, FATHMM score < 0, MetaLR score > 0.5, MetaSVM score > 0, DANN score > 0.8, VEST3 score > 0.75, CADD score > 20, PROVEAN score < -2.5, Mutation Assessor score > 1.9, LRT=D and Mutation Taster=A or D. Finally, we summed the single 12 binary scores to get a total pathogenicity score for each non-synonymous variant. The genetic variants with a total pathogenicity score > 6 were filtered in for further investigation.

In addition, we excluded variants with call rate < 98% as well as those that deviated from the Hardy-Weinberg equilibrium (P < 10^−6^).

### 2.5 Single variant analysis

We performed single-variant analysis (SVA), using a logistic regression model with adjustment for age, as implemented in PLINK 1.9 (https://www.cog-genomics.org/plink/1.9/) (38). To do, we incorporated the ages of the 50 participants as a covariate into the model. Statistical significance was set using Bonferroni correction at 4 × 10^-6^ (0.05/12516 variants tested, after surviving the filtration process). Manhattan plot was generated using CMplot R package (39).

### 2.6 Gene and pathway-based analyses

In order to increase the statistical power, we applied gene and pathway-based tests which combine associations within genes and gene-sets, respectively.

Gene and pathway analyses were conducted using the Multimarker Analysis on GenoMic Annotation (MAGMA) v1.10 software package (40), in a three-step analytic process. First, we mapped the SNPs to genes based on the NCBI 38 human gene reference build. Second, SNP-based EWAS summary statistics, generated by PLINK 1.9, served as input for MAGMA to perform gene-based association analysis. Bonferroni correction was applied for multiple testing correction (p-value = 0.05/2263 genes= 2.2 x 10^-5^). Finally, a competitive gene-set analysis was applied to test for the association of biological pathways with T2D. We used two collections of predefined gene-sets. The first collection was downloaded from the MsigDB v2022.1.Hs database, including Gene Ontology (GO) biological processes, KEGG and Reactome canonical pathways (41, 42)⁠. Significant pathways were called with p-value of 8.99 x 10^-6^ (0.05/5557 gene-sets), according to Bonferroni correction for multiple testing. The second collection consisted of 16 sets of candidate T2D-relevant genes, that were curated by Flannick et al. on their EWAS carried out on 2019 (10)⁠.

### 2.7 Replication analysis

**Figure 1:**
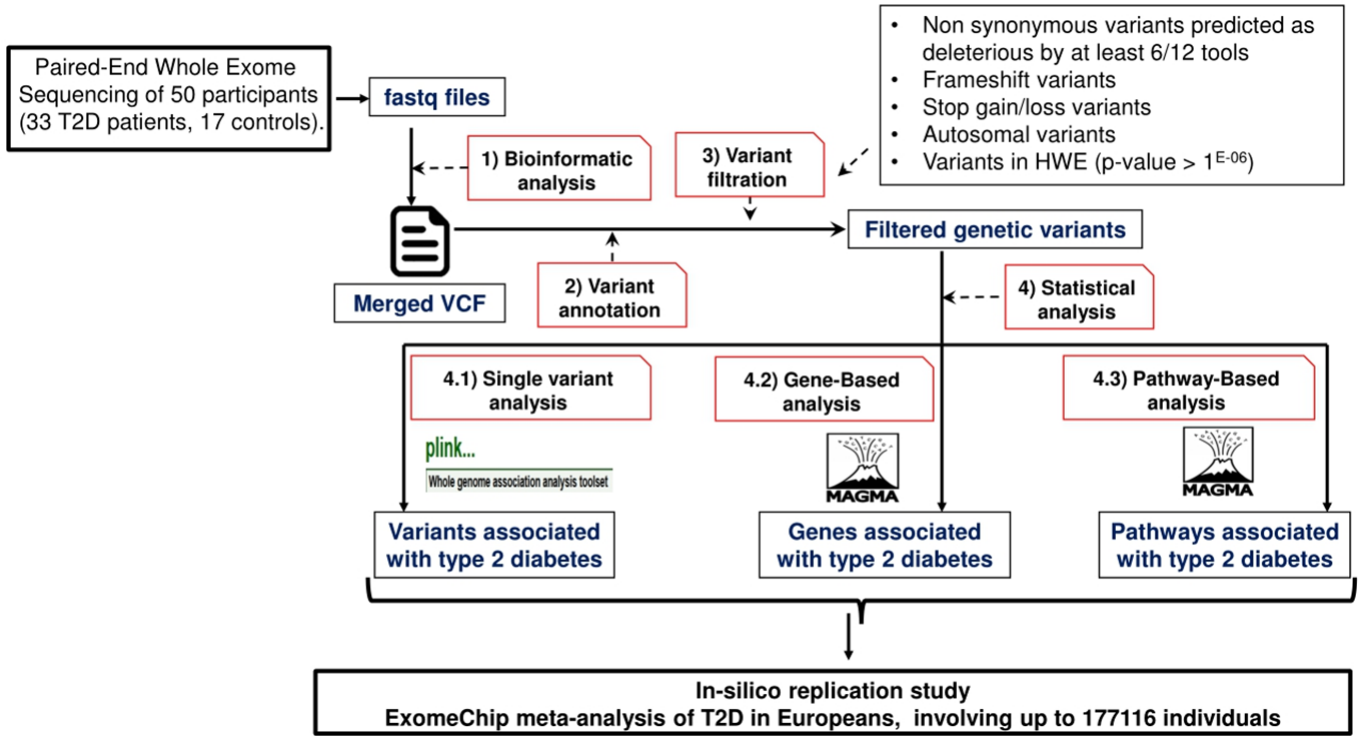
Overview of the study design and statistical analysis pipeline.

We carried out a replication study using the summary statistics of the European ExTexT2D in-silico dataset, downloaded from the DIAGRAM consortium (http://diagram-consortium.org/downloads.html). This dataset consists of ExomeChip meta-analysis of T2D in Europeans, involving up to 177116 individuals, that was previously published in Mahajan et al. (43). We selected a European replication dataset due to the close genetic relationship between Tunisians and Europeans. In fact, previous studies showed that the genetic landscape of the Tunisian population is a mosaic of Eurasian and North African lineages (44–47). This observation may be explained by the migratory waves that occurred in the Mediterranean region since the prehistoric period (44). Gene and pathway-based association p-values were computed with MAGMA using the same code employed in the analysis on the Tunisian subjects. The flowchart of this study is shown in **Figure 1**.

## 3 Results

### 3.1 Characteristics of the study participants

Baseline characteristics of our study participants are summarized in **Table 1**. BMI, fasting plasma glucose and HBA1c were significantly higher in T2D cases compared to controls.

**Table 1:** Demographics of Tunisian type 2 Diabetes cases and controls in the study.

Parameter	Controls	T2D cases	p-value
n	17	33	
Gender (F/M)^1^	12/5	14/19	0.077
Age at examination (yr)^2,3^	46 ± 6	59 ± 6	1.29 x 10^-6^
BMI (kg/m^2^)^2,3^	23.92 ± 3.88	29.12 ± 3.76	0.001
FPG (mmol/L)^2,3^	5.17 ± 0.36	10.22 ± 2.94	1.03 x 10^-8^
HbA1C (%)^2,3^	5.43 ± 0.49	8.95 ± 1.76	4.28 x 10^-8^

^1^ Fisher exact test p-value.

^2^ Data are presented as mean +- standard deviation.

^3^ Wilcoxon rank sum test with continuity correction p-value.

### 3.2 Single variant-association analysis

**Figure 2:**
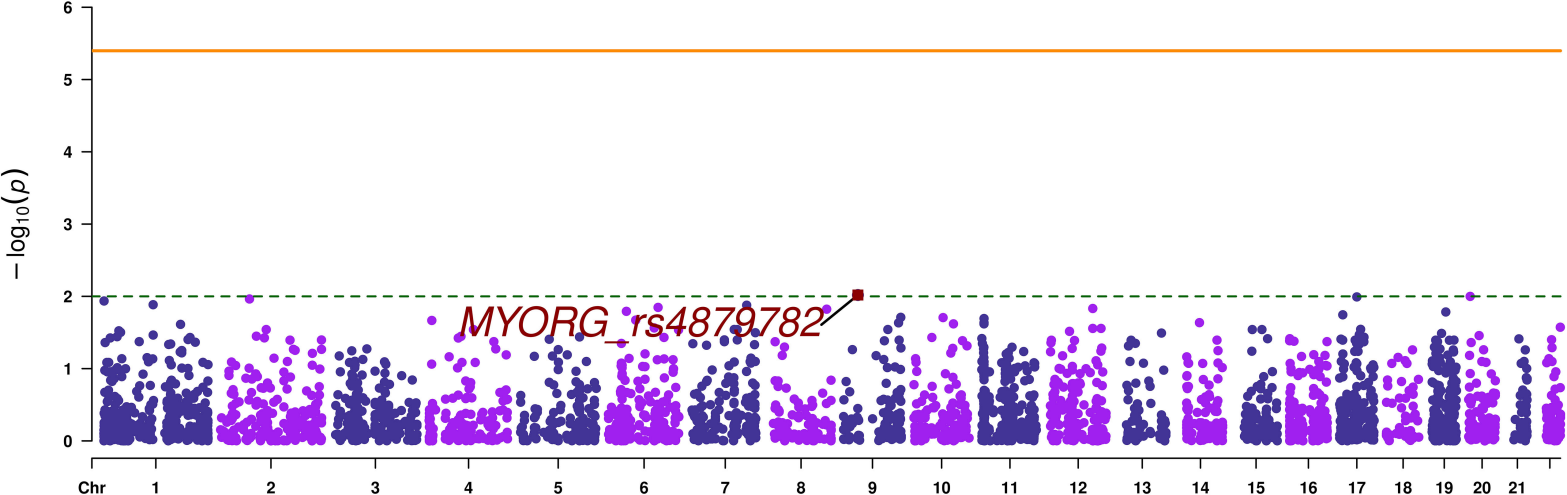
Manhattan plot of single variant analysis. The orange continuous line corresponds to the exome wide significance threshold for single variant analysis (4 x 10^-6^), while the green dashed line corresponds to a nominal association threshold (1 x 10^-2^). The promising variant is highlighted in red.

A total of 12694 variants passed the quality control and variant filtration process to undergo statistical analyses. The Manhattan plot showed that none of the variants reached the exome-significance level (**Figure 2**). The highest peak has an association signal below a nominal threshold of 1 x 10^-2^. This signal stands for the stop-gain variant rs4879782:c.69C>G/p.Tyr23Ter, located in the *MYORG* (Myogenesis regulating glycosidase) gene, with a p-value equal to 9.58 x 10^-3^. However, this signal was not observed in our replication analysis performed on the European in-silico exome-chip dataset. Summary statistics of SVA are provided as **Supplementary Table 1**.

### 3.3 Gene-based analysis

**Figure 3:**
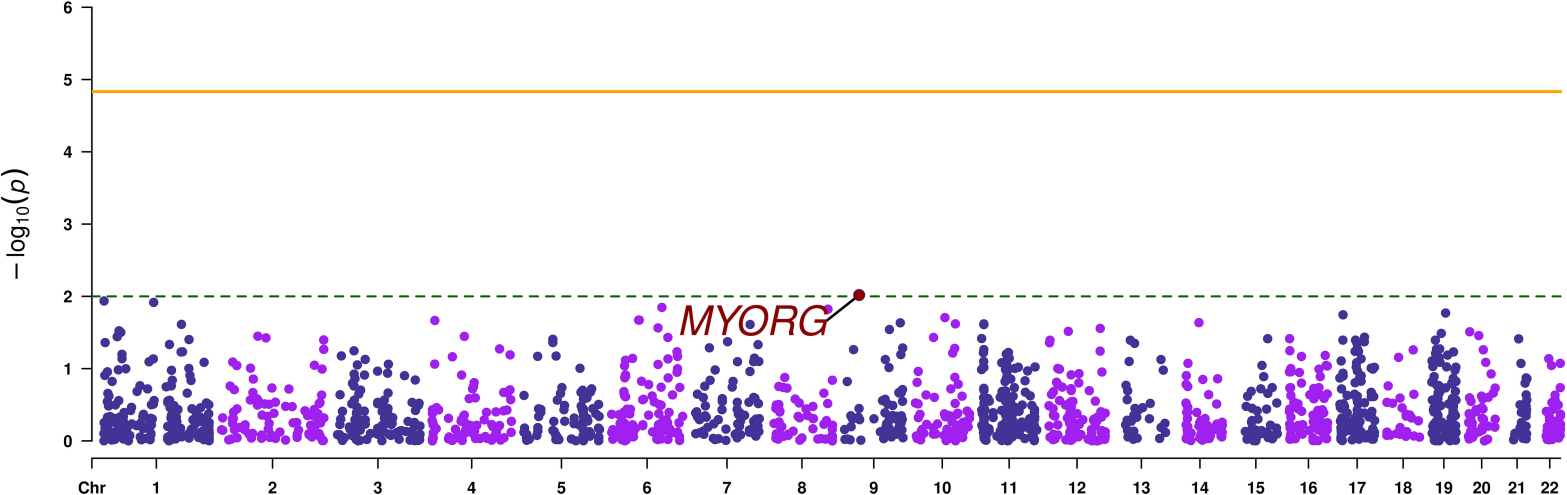
Manhattan plot of the gene-based analysis. The orange continuous line corresponds to the exome wide significance threshold for gene-based analysis (2.2 x 10^-5^), while the green dashed line corresponds to a nominal association threshold (2 x 10^-3^). The promising gene is highlighted in red.

Gene-based analysis using MAGMA, revealed no signals that reached exome-wide significance (p-value < 1.47 x 10^-5^). The top most associated gene was *MYORG* (p-value=9.58 x 10^-3^), as it was the case with the SVA results (**Table 2**; **Figure 3**).

**Table 2:** The results of the gene-based analysis.

Gene	Chromosome	Discovery analysis p-value	Replication analysis p-value
*MYORG*	9	9.58 x 10^-3^	0.39

### 3.4 Pathway-based analysis

We identified two nominal associations of two gene sets from the Gene Ontology database in our study samples (p-value < 2 x 10^-3^). They consist of the positive regulation of neuron apoptotic process and the regulation of mucus secretion (**Table 3**). In addition, our EWAS identified two pathways having nominal association with T2D (p-value < 1 x 10^-2^), both in our study samples and in the European replication dataset (**Table 4**).

**Table 3:** Two candidate pathways that exhibited nominal association with type 2 diabetes in the present study.

Database	Pathway	P-value in the discovery cohort	P-value in the replication dataset
GOBP	Positive regulation of neuron apoptotic process	1.40 x 10^-3^	0.29
GOBP	Regulation of mucus secretion	1.71 x 10^-3^	0.44

GOBP, Gene Ontology Biological Processes.

**Table 4:** Two candidate pathways that exhibited significant association with type 2 diabetes in the present study and in the replication *in-silico* dataset.

Database	Pathway	P-value in the discovery cohort	P-value in the replication dataset
REACTOME	Regulation of beta-cell development	8.30 x 10^-3^	2.32 x 10^-6^
GOBP	Regulation of peptidase activity	6.73 x 10^-3^	5.68 x 10^-3^

GOBP, Gene Ontology Biological Processes.

Regarding pathway analysis using the known T2D-relevant gene-sets, there was not a significant association (all p-values > 0.05).

## 4 Discussion

The clinical heterogeneity of T2D and the development of multisystemic complications suggest that multiple genetic variations are involved in the manifestation of the disease phenotype.

In the present study, we performed a pilot EWAS to interrogate the association of variants, genes and pathways with T2D in Tunisia. The key findings of our study are as follows: 1) single variant and gene-based analyses revealed a promising association signal in *MYORG* gene, although not reaching exome wide significance threshold, 2) gene-set analysis identified two pathways with nominal association to T2D: positive regulation of neuron apoptotic process, and regulation of mucus secretion. However, these pathways’ associations were not replicated in an independent European in-silico exome chip dataset comprising up to 177116 individuals. In addition, our EWAS identified two pathways with nominal association to T2D, both in our study samples and in the European replication dataset.

To date, the majority of high throughput association studies of T2D have been performed in Europeans. There were only a few candidate gene studies of T2D in North African populations, and they yielded conflicting results. For instance, Cauchi et al. reported that 10 among 37 validated European loci were associated with T2D in the Tunisian population (48).

In our pilot EWAS, the SVA and gene-based analysis did not reveal any significant association with T2D in the Tunisian study population. However, they both revealed a promising stop-gain variant, rs4879782, located in the *MYORG* gene. This variant was not previously associated with a clinical condition. On the cellular level, *MYORG* encodes a glycosyl hydrolase, hydrolyzing O-glycosyl compounds in the endoplasmic reticulum. Interestingly, it was reported that *MYORG* is involved in the fiber development of skeletal muscle, the major site for postprandial glucose uptake from the blood circulation (49). Progressive loss of muscle mass is associated with impairment of its roles in regulating glucose homeostasis, which was shown to contribute to the pathogenesis of T2D (50, 51). Based on these observations, further studies are warranted to examine the relationships between *MYORG* mutations and the glucose metabolism.

For pathway-based analysis, we conducted a competitive, regression-based test as implemented by MAGMA (40), to investigate the enrichment of genes associated with T2D.

It is known that T2D is a polygenic trait that is influenced by hundreds of variants across many genes (52). Hence, any random combination of genes is likely to be associated with T2D. We opted for competitive tests that compare the association with T2D of genes in a specific gene-set against genes that are not in this specific gene-set.

Pathway association analysis revealed nominal associations between T2D and two pathways. The first association was with the “positive regulation of neuron apoptotic process”. This pathway is implicated in the maintenance of the neuronal system in adult life by the tight regulation of apoptotic mechanisms. However, excessive activation of this process could lead to pathological conditions such as neurodegenerative diseases (53). Neuronal apoptosis can be triggered through mitochondrial pathways, including the release of caspase proteins or activation of death receptors by the tumor necrosis factor receptor (54). In this context, a recent study has demonstrated excessive activation of caspase and tumor necrosis factor-alpha in T2D rat brains (55). Moreover, it has been shown that apoptosis can be induced by oxidative stress and inflammatory mechanisms triggered by insulin resistance, leading to neurodegenerative diseases such as ischemic stroke and vascular dementia (56, 57). These observations emphasize the relevance of conducting further studies on the effects of genetic alterations in this pathway on the development of T2D neurodegenerative complications. The second association was with the “regulation of mucus secretion” pathway. Intestinal mucus forms a highly organized protective barrier against harmful substances (58). Under perturbed conditions such as infections, pathogens can disrupt the mucus barrier, which facilitates the translocation of microbial products to the epithelial surface. This can cause inflammatory response, which sets favorable conditions for the development of metabolic diseases such as diabetes (59). In addition, a recent study demonstrated that hyperglycemia alters the intestinal barrier, which results in a systemic influx of microbial products, leading to inflammatory consequences of diabetes and obesity (60). In perspective, further studies are needed to decipher the links between the regulation of the gut mucus secretion and the development of metabolic disorders.

Besides these two pathways, MAGMA analysis identified two pathways that showed nominal association (p-value < 1 x 10 ^-2^) with T2D pathogenesis in Tunisians and in a European replication dataset. The first identified pathway was the regulation of beta-cell development. It is known that pancreatic beta-cells ensure the production, the storage and the secretion of the insulin used by the organism to maintain normoglycemia (61). Changes in nutrient state affect the insulin secretory response according to an interplay between extracellular signals and beta cell-specific transcriptional programs. Defects in this interplay process will alter the beta-cells function, leading to a relative insufficiency of insulin blood levels, which is characteristic of T2D (62). Thus, it is undeniable that a deeper understanding of beta-cell transcriptional programs and their associated signals will have direct implications for diabetes therapies. The second pathway was the regulation of peptidase activity. The most known peptidase that affects glucose metabolism is the dipeptidyl peptidase-4 (DPP-4). This protein inhibits the glucagon-like-peptide-1, an incretin expressed in the post-prandial phase in order to stimulate insulin secretion (63). Interestingly, it has been reported that obese and type 2 diabetic subjects have high circulating DPP-4 levels (64). Moreover, it has been proposed that DPP-4 triggers inflammation and insulin resistance in adipose and hepatic tissue (65, 66). These observations promoted the development of DPP-4 inhibitors, used nowadays as oral anti-diabetic medications. However, the overall significance of DPP-4 activity for the regulation of glucose homeostasis remains to be resolved.

A potential limitation of our study resides in the relatively small sample size of the discovery cohort. This limitation may induce a lack of sufficient statistical power to detect all associations. To overcome this limitation, we applied a three-step approach in order to reduce the number of statistical tests, and thus increase the statistical power. First, we prioritized candidate markers that potentially damage protein’s structure and function. Second, we applied gene-based and pathway-based analyses in order to test the aggregate effects of combinations of genetic variants on the regulation of the glycemic homeostasis. Finally, we replicated our analyses in a large existing in-silico dataset. Overall, our strategy led to identify nominal variant and gene associations, as well as biologically relevant pathways associated with T2D in the Tunisian population. Therefore, it seems suitable to discover candidate disease markers in underrepresented populations.

In summary, the present study is the first EWAS on T2D in Africa. Variant and gene-based analyses highlighted changes in *MYORG* gene as new promising markers of T2D in Tunisians, although not reaching exome wide significance. Further gene-set analysis highlighted the potential role of the positive regulation of neuron apoptotic process, the regulation of mucus secretion, beta-cell development and peptidase activity pathways in T2D development and/or progression. These candidate loci and pathways will promote the understanding of T2D pathophysiology in North-African populations. Moreover, they may be the targets for functional studies to discover potential treatments for T2D.

## Data availability statement

The data presented in the study are deposited in the European Variation Archive (EVA) repository, accession number PRJEB70960. Publicly available datasets were analyzed in this study. This data can be found here: http://diagram-consortium.org/downloads.html.

## Ethics statement

The studies involving humans were approved by Comité d’éthique Bio-Médicale de l’Institut Pasteur de Tunis. The studies were conducted in accordance with the local legislation and institutional requirements. The participants provided their written informed consent to participate in this study.

## Author contributions

HD: Conceptualization, Data curation, Formal Analysis, Investigation, Methodology, Resources, Software, Validation, Visualization, Writing – original draft. WB: Data curation, Formal Analysis, Methodology, Resources, Validation, Writing – review & editing. NK: Data curation, Methodology, Resources, Validation, Writing – review & editing. MF: Methodology, Resources, Validation, Writing – review & editing. HJ: Data curation, Methodology, Resources, Validation, Writing – review & editing. MH: Data curation, Methodology, Resources, Validation, Writing – review & editing. IG: Data curation, Methodology, Resources, Validation, Writing – review & editing. MG: Data curation, Methodology, Resources, Validation, Writing – review & editing. WK: Data curation, Methodology, Resources, Validation, Writing – review & editing. MM: Methodology, Validation, Writing – review & editing. MT: Methodology, Validation, Writing – review & editing. AK: Methodology, Validation, Writing – review & editing. HT: Methodology, Validation, Writing – review & editing. AB: Methodology, Validation, Writing – review & editing. HJ: Investigation, Methodology, Resources, Validation, Writing – review & editing. OM: Validation, Writing – review & editing. AA: Investigation, Validation, Writing – review & editing. RK: Conceptualization, Funding acquisition, Investigation, Project administration, Resources, Supervision, Validation, Writing – review & editing.
